# Development of a novel data mining tool to find *cis*-elements in rice gene promoter regions

**DOI:** 10.1186/1471-2229-8-20

**Published:** 2008-02-27

**Authors:** Koji Doi, Aeni Hosaka, Toshifumi Nagata, Kouji Satoh, Kohji Suzuki, Ramil Mauleon, Michael J Mendoza, Richard Bruskiewich, Shoshi Kikuchi

**Affiliations:** 1 National Institute of Agrobiological Sciences, 2-1-2 Kannondai, Tsukuba, Ibaraki 305-8602, Japan; 2 Hitachi Software Engineering Japan Co., Ltd., 6-81 Onoe-cho, Naka-ku, Yokohama 231-0015, Japan; 3 International Rice Research Institute, DAPO 7777, Metro Manila, Philippines

## Abstract

**Background:**

Information on more than 35 000 full-length *Oryza sativa *cDNAs, together with associated microarray gene expression data collected under various treatment conditions, has made it feasible to identify motifs that are conserved in gene promoters and may act as *cis*-regulatory elements with key roles under the various conditions.

**Results:**

We have developed a novel tool that searches for *cis*-element candidates in the upstream, downstream, or coding regions of differentially regulated genes. The tool first lists *cis-*element candidates by motif searching based on the supposition that if there are *cis-*elements playing important roles in the regulation of a given set of genes, they will be statistically overrepresented and will be conserved. Then it evaluates the likelihood scores of the listed candidate motifs by association rule analysis. This strategy depends on the idea that motifs overrepresented in the promoter region could play specific roles in the regulation of expression of these genes. The tool is designed so that any biological researchers can use it easily at the publicly accessible Internet site . We evaluated the accuracy and utility of the tool by using a dataset of auxin-inducible genes that have well-studied *cis-*elements. The test showed the effectiveness of the tool in identifying significant relationships between *cis-*element candidates and related sets of genes.

**Conclusion:**

The tool lists possible *cis-*element motifs corresponding to genes of interest, and it will contribute to the deeper understanding of gene regulatory mechanisms in plants.

## Background

With the completion of rice genome sequencing by the International Rice Genome Sequencing Project [1], the Beijing Genomics Institute (BGI) [2], and Syngenta [3], many rice functional genomic resources have become available, including whole genome sequences from ssp. *japonica *'Nipponbare' and ssp. *indica *line 93-11; a set of rice full-length cDNA clones and their complete and partial end sequences [4,5], microarray gene expression systems based on full-length cDNA sequences, ESTs (Expressed Sequence Tag), MPSS (Massively Parallel Signature Sequencing), SAGE (Serial Analysis of Gene Expression), and predicted genes in the genome sequences; and many kinds of insertion mutants with Tos17, Ac-Ds, and T-DNAs [6]. As analytical technology progresses, the database continues to be upgraded and serves as a useful resource for studying mechanisms that regulate gene expression.

*Cis-*elements in the promoter regions of genes and *trans-*acting transcription factors are major biological features to be characterized if we are to achieve an understanding of the systems that regulate gene expression. Identification of candidate *cis-*elements corresponding to genes is now practicable through the use of available sequence and genome mapping information, combined with information about the responses of genes to specific experimental conditions; such responses have been elucidated by using gene expression profiles now publicly available.

Exhaustive sequence analysis by using available public databases can identify *cis-*element candidate motifs for further examination, but such approaches are not quite efficient. One confounding factor is that public databases are independently constructed and not generally optimized to facilitate integration of information from many sources with local experimental data. A more perplexing issue for experimental researchers who are not very familiar with bioinformatics techniques is the challenge of finding unknown but biologically notable relationships among genes, *cis-*elements, and experimental conditions from the huge number of possible combinations generated by large experimental data sets.

To resolve some of these issues, we developed a novel data mining tool to identify *cis-*elements in the rice genome. It performs the complex bioinformatics analysis mentioned above, then lists *cis-*element candidates for genes. The genes can be grouped by similarity of expression profiles and other criteria for assessment by researchers, then the tool annotates them with related public database information.

Similar tools have been developed previously. Helden released RSAT, which includes a program that can detect over-represented motifs in upstream regions of co-regulated genes [7]. Holt et al. established CoReg, which links the hierarchical clustering of co-expressed gene sets with frequency tables of promoter elements [8]. Zhao et al. established TRED, which integrates a database and a system for predicting *cis*- and *trans*-elements in mammals [9]. Galuschka et al. developed AthaMAP, which includes a program for comparative analysis of *cis*-elements in sets of co-transcribed genes of *Arabidopsis thaliana *[10].

Our tool is distinguished by several points: (i) It focuses on the rice genome, being based on full-length cDNAs, and is designed to pick up *cis*-element candidates associated with genes that users designate. (ii) It evaluates the likelihood score of *cis*-element candidates by comparing frequency counts in the user-selected gene set and a reference gene set. (iii) It can evaluate previously known *cis*-element sequences as well as user-specified sequences prepared by other analysis tools, and it can examine several *cis*-elements together.

The tool carries out both *ab initio *motif searches of promoter sequences and searches against known plant *cis-*elements, then performs a likelihood analysis of identified *cis-*elements on the basis of their presence in a significant proportion of the promoters of a given set of genes. This evaluation is achieved by an association rule analysis.

Here, we present technical details of the tool and demonstrate the practical assessment of its utility with a biologically relevant sample data set.

## Implementation

**Figure 1:**
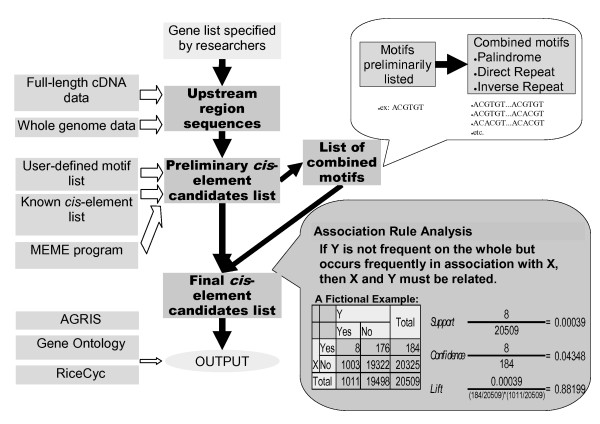
Features of RiCES.

The tool, called Rice *Cis*-Element Searcher (RiCES), consists of a *cis-*element searching pipeline, controlled via a Web-based user interface. Fig. 1 summarizes the procedure. The pipeline first reads a list of gene identifiers from the user, which it uses to retrieve the promoter sequences corresponding to the listed genes. Then a preliminary list of *cis-*element candidates is built by aligning information from the built-in list of plausible motifs, or by *ab initio *motif searching of the sequence data. Association rule analysis is carried out and reported to support the candidacy of the resulting *cis-*element list.

### Gene list

RiCES assumes that a user has already identified genes of interest from experimental analysis (e.g. clusters of coordinately regulated genes). The list of identifiers is input into a Web-based data entry form. RiCES recognizes GenBank accession numbers, identifiers of transcription units (TUs) as defined in the TIGR pseudomolecular assemblies [11], and several other major gene identification systems. Using the list, it retrieves the set of associated upstream, downstream, or coding region sequences flanking the specified genes from available genomic sequence data.

### Preliminary *cis*-element candidate list

The second step of the analysis is the compilation of a list of motifs as candidate *cis-*elements. At present RiCES supports two methods to achieve this.

The first method depends on *ab initio *motif searching based on the supposition that if there are *cis-*elements playing important roles in the regulation of a given set of genes, they will be statistically overrepresented in the associated promoter sequences as conserved motifs that can be identified by using a suitable motif search program. There are several programs implementing several algorithms. We have chosen to use MEME, which is a publicly available motif discovery program [12] supporting an expectation maximization algorithm. In our analysis algorithm, MEME is invoked to identify motifs 6 to 8 bp long that look highly conserved among promoter sequences of the selected genes. Users can modify some of the search parameters of the MEME program via the Web form.

The second method relies on the hypothesis that common, known *cis-*elements play important roles under the experimental conditions that gave rise to the list of genes specified by the user. Therefore, RiCES searches for matches to a pre-compiled list of known *cis-*elements.

Several databases of plant *cis-*elements are publicly available. PLACE [13] is one of the most popular databases of known *cis-*elements in plant genomes. AtcisDB, a part of AGRIS [14], includes information on *cis*-elements involved in gene regulation in *Arabidopsis thaliana*.

Although these databases are extremely useful resources, it is not straightforward to cross-link information from them directly to the researcher's own data. Current databases are not exhaustive enough to distinguish 'core' motifs, which decide the function of *cis*-elements, from co-existing sequences in neighboring regions. As a result, many *cis*-element sequence data in these databases include superficial core motifs for which no evidence of functionality has been obtained. The use of such data prohibits effective informatic analysis.

We compiled a novel database of known *cis-*elements and incorporated it into RiCES [See Additional file 1]. The *cis-*elements are collected from reports of experiments such as gel shift assays and footprint analyses, categorized by transcription factor, and documented with respect to known activity in the plant genome. Some *cis-*elements known only in organisms other than plants are also listed, in consideration of their possible, albeit unknown, roles in plants. The database includes four types of *cis*-elements: (1) G-box and E-box, which bind to common sequences such as bHLH or bZIP in many organisms; (2) A-box, T-box, and GGTTTAG repeats, which bind to common sequences in many organisms, such as homeodomain and Myb; (3) CArG boxes and GCC-box, which bind to plant MADS, zinc finger, and AP2/EREBP elements; and (4) other *cis-*elements, binding only in animals, such as HSF, PcG, and HMG.

### Association rule analysis

The third step of the analysis is the likelihood evaluation of the *cis-*element candidates by association rule analysis, which is a data mining method designed to discover significant relationships between pairs of characteristics observed in data sets. Candidates showing the highest likelihood (specificity) are retained in the final *cis-*element candidate list.

Association rule analysis has been applied to mechanisms that regulate gene expression [e.g. [15,16]]. We used it to find relationships between identified *cis-*elements and gene expression profiles. The strategy depends on the idea that motifs overrepresented in the promoter region of the genes of interest could play specific roles in regulation of the expression of those genes.

Implied cause-and-effect relationships documented as 'rules' are evaluated by using several well-known indices of likelihood, including *support*, *confidence*, and *lift *[15]. On the basis of sample data sets, the *lift *index appeared to best discriminate significant relationships between experimental conditions and *cis-*element candidates.

In a rule described as

*the presence of motif X in a gene implies that the gene is a member of group Y*,

*lift *is the ratio of the posterior probability (the probability that the gene is in group Y if it possess motif X) to the prior probability (the probability of X possession, irrespective of the membership of Y). When *lift *> 1.0, the coexistence of X and Y is not a random occurrence, but suggests some causal relationship between them. If *lift *< 1.0, it is not considered probabilistically significant. Consequently, we set the default threshold of *lift *to 1.0, and the *cis-*element candidates are included in the final candidate list only if their *lift *value is higher than this threshold.

RiCES also evaluates pairwise combinations of motifs in the preliminary candidate list (upper right-hand box in Fig. 1), in consideration of possible protein-protein interactions of multiple transcription elements binding *cis-*elements, as illustrated by experimental evidence [17,18].

### Output

The final *cis-*element candidate list is presented as an association table with the identifier of the submitted genes (TU identifiers based on TIGR gene model annotation are used in the current version) annotated with any available corresponding information from RiceCyc [19] and Gene Ontology [20]. RiCES also provides information on candidate motifs, including the positions of the element in the promoter regions of corresponding TUs, the sequence, and related information from AtcisDB [14]. The position of the *cis-*element candidates is also presented in both text and graphics.

## Validation

To test whether or not the output of RiCES was meaningful, we validated it with a list of auxin-inducible genes with known characteristics, compiled from RiceTFDB 2.0 [21]. First, Aux/IAA genes stored in RiceTFDB were applied as queries in a BLASTN search [22] of GenBank, returning a list containing 28 rice TUs [See Additional file 2]. These genes were fed into the pipeline. When the MEME program was called, the length of target motifs was set to 6, 7, or 8 bases, the number of occurrences of each motif was set to 7, 14, or 21, and the search algorithm was set to 'zoops' to check zero or one occurrence per sequence. The outputs of each option setting were merged but not otherwise filtered.

## Results and Discussion

Many Aux/IAA genes are auxin-inducible [23] and contain the TGTCTC element [24]. This element is commonly found in the upstream region of auxin-responsive genes. Thus, the detection of all instances of the motif by the pipeline could serve as a validation of the pipeline algorithm. The auxin-responsive element (AuxRE) containing the TGTCTC motif in some cases requires another proximal AuxRE for biological activity [17,25]. In other contexts, AuxRE functions only when it occurs with its palindromic components separated by 7 or 8 nucleotides [26].

In our validation test, MEME listed 7514 motifs in total from 1000 bp of the upstream sequences [See Additional file 3], of which 4128 showed a high *lift *value (>1.0) [See Additional file 4]. A search of AtcisDB for these motifs returned 4 showing a partial match to the record of 'PRHA binding sites' (Table 1), which is derived from the report of Plesch et al. [27], describing auxin-induced expression of the *Arabidopsis prha *homeobox gene. Another 4 motifs contained the TGTCTC element. The result was consistent with previous work, as TGTCTC was listed as a candidate in the single motif search of Aux/IAA genes.

**Table 1:** Cis-element candidate motifs from Aux/IAA genes and suggested to be auxin-induction related according to ATCIS.

Motif	Hit TU in target group*^1^	Hit TU in whole*^2^	Lift	ATCIS Description
ACACAC	10	6056	1.353	PRHA BS in PAL1*^3^
ATACACA	5	2124	1.929	PRHA BS in PAL1
ATACACAC	3	739	3.326	PRHA BS in PAL1
TACACAC	4	1786	1.835	PRHA BS in PAL1
CATGTCTC	1	303	2.704	-
GTGTCTC	1	722	1.135	-
TGTCTCCG	1	178	4.603	-
TGTCTCTG	2	263	6.231	-

*1 The number of TU possessing the designated motif within 28 TUs of the target gene list.

*2 The number of TU possessing the designated motif within 22943 TUs stored in KOME database.

*3 PRHA = Developmental and auxin-induced expression of the Arabidopsis prha homeobox gene.

Table 2 shows the result of the validation test with a pre-compiled *cis-*element list generated by the test gene list. The analysis returned 22 *cis*-element candidates with *lift *> 1.0 [See Additional file 5 and 6]. Some of these candidates were suggested by previous studies to have some kind of relationship to auxin response. For example, *RAV1 *was found in the promoter region of *ABP*, which encodes an auxin-binding protein [28]. Expression of *LEAFY *(*LFY*) is affected by the auxin gradient in *Arabidopsis *[29]. *ETT *is another auxin response factor [30], and *LFY *and *ETT *expression are closely correlated [18,31].

**Table 2:** Cis-element candidates selected from the pre-compiled list, likely corresponding to Aux/IAA genes.

Motif	Transcription Factor Family*^1^	Hit TU in target group*^1^	Hit TU in whole*^2^	Lift
([ACGT]GAA [ACGT]){3}	HSF	4	512	6.40
TGACAGGT	Helix-turn-helix(HTH)	3	527	4.66
CCAC [AC]A [ACGT] [AC] [ACGT] [CT] [AC]	LIM finger	9	3013	2.45
GG [ACGT]CCCAC	Helix-loop-helix factors(bHLH)	10	3601	2.28
GTGG [ACGT]CCC	Helix-loop-helix factors(bHLH)	6	2189	2.25
CAACA [ACGT]*CACCTG	RAV	5	1865	2.20
A [TC]G [AT]A [CT]CT	EIL	8	3039	2.16
AATATATTT	Helix-turn-helix(HTH)	3	1405	1.75
TGTCTC	ARF	7	3825	1.50
TGACGTGG	NAC	1	627	1.31
CCA [ACGT]TG	LEAFY	19	12084	1.29
CACCC	Cys2His2 zinc finger;RING finger	19	12165	1.28
CC [AT]{6}GG	MADS(CArG boxes)	2	1392	1.18
AATAAA [CT]AAA	Helix-turn-helix(HTH)	1	715	1.15
CGTG [TC]G	BZR(BES1)	9	6544	1.13
[GC] [GC] [GA]CGCC	BRE	10	7543	1.09
AGCCGCC	EREBP	2	1523	1.08
CCAAT	CCAATbox;Co-like	19	14497	1.07
TATA [AT]A	TATAbox	22	16849	1.07
[TA]AAAG	Dof	27	21329	1.04
CA [ACGT] [ACGT]TG	Helix-loop-helix factors(bHLH);Helix-loop-helix_leucine zipper factors(bHLH-ZIP)	28	22405	1.02
T{4,6}	JUMONJI	28	22699	1.01
[CT] [CT]A [ACGT] [TA] [CT] [CT]	Inr	28	22899	1.00
(GA){2,}| (TC){2,}	BBR/BPC	28	22911	1.00

*1 The number of TU possessing the designated motif within 28 TUs of the target gene list.

*2 The number of TU possessing the designated motif within 22943 TUs stored in KOME database.

The position of a *cis-*element is important information to consider in relation to the function of the *cis-*element. For biological activity to occur, the distance of some *cis-*elements from the coding region or other collaborating elements is constrained. To this end, RiCES highlights the distribution of *cis-*element candidates. It provides tables of identified *cis-*element motifs and graphical motif maps to help researchers grasp positional relationships among the candidate elements.

**Figure 2:**
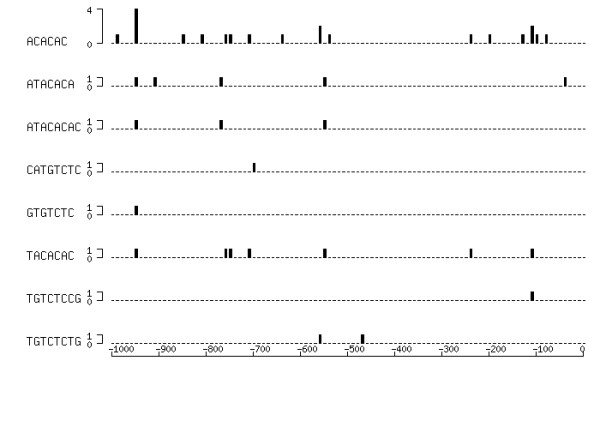
**Distribution of the 15 Aux/IAA-related *cis*-element candidates. **The presence of the motifs of candidates with high *lift *values (see 4th column in Table 1) was searched in the 1000-bp upstream region of genes, and frequency was counted in segmented regions at an interval of 10 bp. The X-axis represents the position in the upstream region, and the bars designate frequency of motifs (counted after distribution of multiple regions was merged).

The positions of the listed elements, some of which include TGTCTC, varied among upstream regions of genes (Fig. 2), and it was hard to detect any skewed distribution of motifs. Goda et al. [32] studied the distribution of TGTCTC motifs in the genome of *A. thaliana*, and pointed out that 25% of investigated genes had TGTCTC motifs in the upstream region within 1000 bp of the start codon, and 14% within 500 bps. Our results do not seem in conflict of theirs.

TGTCTC motifs are scattered over wide regions of many plant species (Table 3). It is possible that the variety of the roles of genes reflects the variety of mechanisms regulating gene expression and positions of *cis-*elements, even if the genes in question can be classified as 'auxin-responsive genes' in a larger sense.

**Table 3:** Representative plant genes possessing TGTCTC element in corresponding upstream region.

Gene (domain)	Position	Remarks	References*
GH3 (D4)	-130~-125	The auxin-responsive soybean GH3 gene. Domain D4 and D1.	[17, 33]
GH3 (D1)	-176~-171		
OsBLE3	-434~-429	Brassinolide-enhanced gene involved in cell elongation in rice through dual regulation by BL and IAA.	[34]
GhMyb7	-75~-70	A cotton R2R3-MYB gene. The transcript level is increased by auxin in fiber cells in an in vitro ovule culture system.	[35]
PsPK2	-1695~-1690	PINOID-like gene from *Pisum sativum*. Auxin and gibberellin positively regulate its expression.	[36]
14-3-3	-625~-620, -531~-526	Promoter of the gene of 14-3-3 proteins, participating in cell cycle control, was investigated in *Solanum tuberosum*.	[37]
LCA1	-1430~-1425	Ca2+ -ATPase gene of *Lycopersicum esculentum *induced by ABA and IAA.	[38]
CMe-ACS2	-106~-101	ACS (auxin-responsive 1-aminocyclopropane-1-carboxylate synthase gene) of Melon (*Cucumis melo*).	[39]
OsRAA1	-150~-145	OsRAA1(*Oryza sativa *Root Architecture Associated 1) functions in the development of rice root system.	[40]
PsNin	-364~-359	Genes function in early stages of root nodule formation in *Pisum sativum *(PsNin) or in *Lotus japonicus (LjNin)*.	[41]
LjNin	-365~-360		
SAUR	-134~-129	SAUR (Small Auxin-Up RNA) gene of *Glycine max*.	[42]
CEVI1	-959~-954, -119~-114	Defense-related CEVI1 gene is found from tomato(*Lycopersicon esculentum*).	[43]
EXPA1	-2090~-2085	TSI (tropic stimulus-induced) genes observed in *Brassica oleracea*.	[44]
SKS1	-1204~-1199		
SAUR50	-101~-96		
GH3.5	-86~-81, -585~-580		
AAP8	-918~-913	*Arabidopsis *amino acid transporters (AAPs); AAP8 is probably responsible for import of organic nitrogen into developing seeds.	[45]

*) Numbers are equivalent to those shown in the main text.

A major research concern is how to pick up *cis-*element candidates worthy of further experimentation. Computational and manual selection of *cis-*element candidates should play complementary roles to resolve this issue. It should be emphasized that *cis-*element candidates listed by RiCES are rated according to the likelihood provided by association rule analysis. On the other hand, researchers can check the significance of candidates in detail by using related information derived from several databases. The supported databases include AGRIS, Gene Ontology, and RiceCyc, as well as the map information described above.

**Figure 3:**
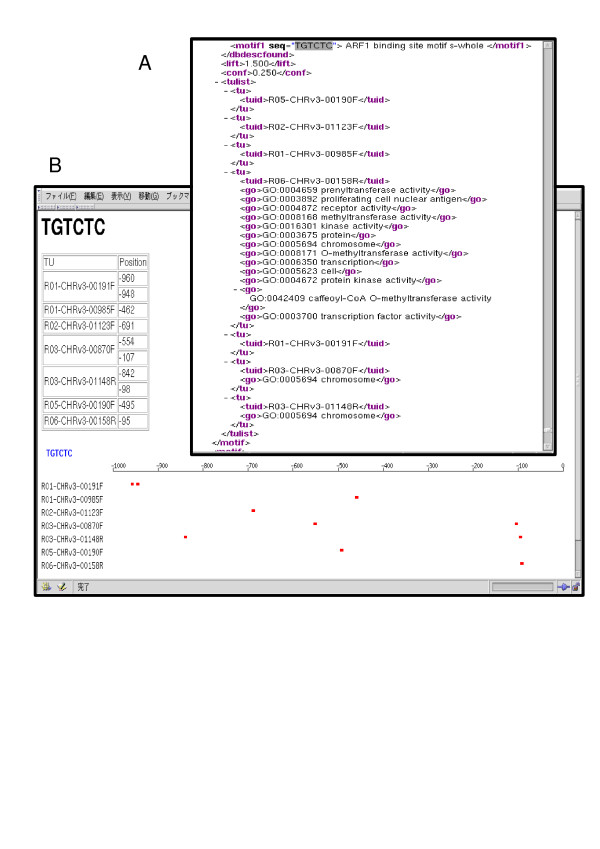
**Snapshots of representative outputs of RiCES.** A: List of *cis*-element candidate motifs including related information. B: Mapping image of *cis*-element candidate motifs.

Fig. 3 is an example of the output for the TGTCTC motif. The outputs are not only easily accessible in a Web browser, but are also usable in further statistical or bioinformatics analysis, as they are also provided in XML format (Fig. 3A), which is a tagged plain-text format compatible with various computer programs.

In some cases, the results of the analysis from the pre-compiled list of elements will be easily comparable with prior knowledge. In other cases involving solely *ab initio *evidence from MEME, the results of motif searches should be interpreted carefully, because the result will change considerably in accordance with the options selected. An appropriate set of motif search options should be determined each time, by trial and error. However, as described above, a motif search can find *cis-*element candidates of which the sequences do not exactly match those of known *cis-*elements.

Although RiCES is focused on the role of *cis-*elements in *Oryza sativa *ssp. *japonica*, the methodology can be applied easily to studies of other plant species, or of other genome sequence motifs involving gene expression regulation, such as motifs in coding regions of genes or downstream of the gene sequence. Such work can be made possible by replacing the reference data set containing whole genes of rice with other data sets.

## Conclusion

We presented here a newly developed tool to search for *cis-*element candidates in a list of genes. A case study showed the applicability of the tool. The tool is easy to use and publicly available. We expect that its use will deepen understanding of the mechanisms that regulate gene expression in plants.

## Availability and requirements

RiCES is accessible at  by any JavaScript-capable browsers.

**Project Name: **Generation Challenge Programme Subprogramme 4

**Project Home Page**: 

**Operating system(s): **Platform independent

**Other requirements: **None

**Programming language: **Perl

**License: **Freely available for use

**Any restrictions to use by non-academics**: None

## Authors' contributions

KD designed the algorithm, did all the programming, and performed the feasibility test of the tool. AH helped to prepare test data sets and the literature search. TN supplied the inner database of known *cis*-elements to which the tool refers. KSa and KSu prepared the reference data. RM, MJM, and RB made many technical suggestions on the implementation and set up the host computer. RB also corrected the English of this manuscript. SK conceived the study and participated in its design and coordination. All authors read and approved the manuscript.

## Supplementary Material

Additional file 1 Known plant *cis*-elements listed for analysis by RiCES. See text for further details. Click here for file

Additional file 2 Transcription units (TUs) used in the feasibility test. Auxin-inducible genes were picked up from RiceTFDB 2.0 (1st column). Corresponding full-length cDNAs were designated by BLASTN (2nd column) and translated to TUs defined in Pseudomolecule ver. 4 (3rd column). Click here for file

Additional file 3 Preliminary list of *cis*-element candidates listed by MEME analysis for TUs shown in Supplementary Table S2. See text for further details. Click here for file

Additional file 4 Result of association rule analysis of *cis*-element candidates listed by MEME. 1st column: examined sequence. 2nd column: number of TUs possessing the designated motif within 28 TUs of the target gene list. 3rd column: number of TU possessing the designated motif within 22 943 TUs stored in KOME database. 4th column: *lift *value. Click here for file

Additional file 5 Result of sequence search for motifs shown in Supplementary Table S1 in 22 943 TUs stored in KOME database. 1st column: examined TUs. 2nd column: motifs found in upstream region of TU. Other columns: position of motifs within the upstream region of each TU. Click here for file

Additional file 6 Result of association rule analysis after sequence search shown in Supplementary Table S6. 1st column: examined sequence. 2nd column: number of TUs possessing the designated motif within 28 TUs of the target gene list. 3rd column: number of TU possessing the designated motif within 22 943 TUs stored in KOME database. 4th column: *lift *value. Click here for file
